# Molecular basis of extreme resistance in *Plasmodium falciparum* to atovaquone and other mitochondrial inhibitors

**DOI:** 10.1186/1475-2875-13-S1-P81

**Published:** 2014-09-22

**Authors:** Sasha Siegel, Andrea Rivero, Dennis Kyle

**Affiliations:** 1 University of South Florida, Tampa, Florida, USA

## Background

Atovaquone is a safe, effective antimalarial drug that inhibits the mitochondrial electron transport chain (mtETC) of *P. falciparum*. Clinical resistance is conferred by SNPs in cytochrome b. Although multiple non-synonymous SNPs can be selected *in vitro*, thus far clinical resistance is limited to amino acid substitutions at position 268 (e.g., Y268S). We are investigating additional mechanisms by which some isolates of *P. falciparum* became extremely resistant to atovaquone and other mtETC inhibitors by selecting for *in vitro* resistance to the dihydroorotate dehydrogenase (DHODH) inhibitor DSM-1.

## Methods

We characterized isolates from patients that failed treatment in atovaquone phase II studies by using a series of chemotypes that target mitochondrial function. We defined their structure-activity relationships and observed broad resistance (5-10,000 fold in atovaquone), suggesting that cytochrome b mutations alone are not sufficient. We tested this by selecting for DSM-1 resistance in atovaquone-susceptible and resistant parasites (paired patient admission and recrudescent isolates). Following selection, the parasites were sequenced for mutations in cytochrome b, DHODH and other candidate mtETC genes.

## Results

The extreme atovaquone resistance phenotype accompanies a high-grade resistance to all mtETC inhibitors tested including DSM-1. Selection studies of atovaquone susceptible parasites rapidly generated resistance *in vitro* to both atovaquone and DSM-1 alone (10x IC_50_ concentration) yet atovaquone resistant parasites could only generate low resistance to DSM-1 (2x IC_50_), and one population reverted the aa268 mutation to wild-type to cope with DSM-1 pressure. Furthermore, we successfully recapitulated the clinical Y268S mutation *in vitro* in TM90-C2A, the pre-treatment parent for TM90-C2B.

## Conclusions

Cytochrome b mutations are not sufficient to explain extreme mtETC resistance, and selective pressure on the DHODH enzyme revealed that the Y268S cytochrome b mutation incurs a fitness cost on the parasite that cannot be overcome under DSM-1 drug pressure, yet resistance was easily obtained in atovaquone-susceptible parasites. This suggests that resistance to atovaquone (Y268S) and DSM-1 are mutually incompatible. Selection of the Y268S mutation in TM90-C2A indicates that the parasite genetic background is critical in the selection of clinical atovaquone resistance, since selection attempts in multiple other strains results in mutations in positions other than aa268. Further studies are required to determine the impact of this phenotype on the development of new mitochondria-targeted drugs.

